# Differences in Clinical Features and Laboratory Results between Adults and Children with SARS-CoV-2 Infection

**DOI:** 10.1155/2020/6342598

**Published:** 2020-12-01

**Authors:** Xiaoli Li, Yan Rong, Peiyan Zhang, Junli Wang, Liping Qie, Lei Rong, Jian Xu

**Affiliations:** ^1^Department of Respiratory and Critical Care Medicine, Shenzhen Hospital, Southern Medical University, Shenzhen 518101, China; ^2^Department of Infectious Diseases, Shenzhen Third People's Hospital, Second Hospital Affiliated to Southern University of Science and Technology, Shenzhen 518000, China; ^3^Department of Pediatrics, Shenzhen Hospital, Southern Medical University, Shenzhen 518101, China

## Abstract

Severe acute respiratory syndrome coronavirus 2 (SARS-CoV-2) infection in children accounts for a small proportion of all infections and is usually mild or asymptomatic. There are few studies on the clinical characteristics of SARS-CoV-2 infection in children, and the causes of the low prevalence in children remain unclear. Herein, we compared the epidemiological and clinical characteristics of SARS-CoV-2 infection between adults and children. Fifty-two patients with Coronavirus Disease 2019 (COVID-19) were retrospectively analyzed, including 38 adults and 14 children. Their clinical information such as epidemiological exposure history, laboratory indicators, chest computed tomography (CT) performance, and number of SARS-CoV-2 positive days were analyzed and compared. In children, 5 (35.71%) had mild COVID-19 and 9 (64.29%) had common type, while, in adults, 9 (23.68%) cases were mild, and 29 (76.32%) were common COVID-19. Among them, family clustering infection accounted for 50% (7/14) of child cases and 23.68% (9/36) of adult cases. Epidemiological exposure history, clinical classification, clinical symptoms, chest CT manifestations, and number of SARS-CoV-2-positive days were not significantly different between children and adults. However, the percentage of neutrophils in adults was significantly higher than that in children (*P* < 0.05). The percentage and absolute value of lymphocytes, platelet counts, aspartate aminotransferase, and aspartate aminotransferase/alanine aminotransferase in adults were lower than those in children (*P* < 0.05). Conclusively, children infected with SARS-CoV-2 show the characteristics of family clustering, and the proportion of mild and asymptomatic infections is higher. For families with a history of epidemiological exposure, routine SARS-CoV-2 nucleic acid testing and chest CT examination should be performed in asymptomatic children to determine whether they are infected. Unlike adults, although the reduction of lymphocytes and platelets in children is not common, it is necessary to be alert to the increased risk of liver damage in children.

## 1. Introduction

Coronavirus Disease 2019 (COVID-19), caused by severe acute respiratory syndrome coronavirus 2 (SARS-CoV-2), is highly contagious and has spread widely around the world. As of June 8, 2020, SARS-CoV-2 has caused approximately 6.8 million infections and more than 380,000 deaths worldwide. However, the proportion of SARS-CoV-2 infection and death in children is relatively low. A survey of 45,000 COVID-19 patients in China showed that about 98% of the infected people were adults, and the remaining 2% were children of 1-19 years old [1]. Similarly, among the confirmed patients reported in the United States, the population under the age of 18 accounted for only 1.7% [2]. In addition to SARS-CoV-2, during 2002-2003 SARS-CoV and 2012 MERS (Middle East Respiratory Syndrome Coronavirus) epidemic, children's risk of coronavirus infection was also low (SARS was about 2%, while MERS was even lower) [3, 4]. Compared with adults, children usually have mild and asymptomatic SARS-CoV-2 infection, with lower viral load [5]. In addition, the symptoms of SARS-CoV-2 infection in children are different from those in adults, which are mostly characterized with fever, cough, or shortness of breath [6, 7].

The S protein of SARS-CoV-2 binds to the angiotensin-converting enzyme (ACE2) receptor on respiratory tract epithelial cells, and thus, the virus can enter the cells [8]. As the immune system gradually matures with age, the expression of ACE2 receptors also increases [9]. This could explain the lower number of SARS-CoV-2 infections in children. The ACE2 expression is higher in SARS-CoV-2-infected children as compared with noninfected children [10]. At present, there are few reports on the clinical characteristics and epidemiology of SARS-CoV-2 infection in children.

In this study, we retrospectively compared the differences between children and adult patients with COVID-19. Their clinical characteristics, number of SARS-CoV-2 positive days, laboratory results, and chest computed tomography (CT) performance were analyzed.

## 2. Materials and Methods

### 2.1. Study Design and Subjects

This retrospective study was approved by the Ethics Committee of Shenzhen Hospital of Southern Medical University, Shenzhen, China. The data were anonymous, and informed consent was therefore waived. We included 52 patients who were diagnosed with COVID-19 from February 1 to March 20, 2020, and were hospitalized in Shenzhen until 2 consecutive SARS-CoV-2 nucleic acid negative tests results were obtained. Patients were then transferred to Shenzhen Hospital of the Southern Medical University. Among them, 14 were children and 38 were adults. The COVID-19 was diagnosed based on “Diagnosis and Treatment of Pneumonia Caused by Novel Coronavirus (Trial Version 5)” and “Diagnosis, Treatment, and Prevention of 2019 Novel Coronavirus Infection in Children: Experts' Consensus Statement” [11, 12]. The inclusion criteria were as follows: nasal/pharyngeal swab tested positive for SARS-CoV-2 nucleic acid, epidemiological and clinical history, and positive results for SARS-CoV-2-specific IgM and IgG antibodies. The exclusion criteria were as follows: negative SARS-CoV-2 nucleic acid test; patients with dysfunction of the heart, liver, kidney, or brain. For clinical classification, asymptomatic cases were individuals infected by SARS-CoV-2 who remain asymptomatic throughout the course of the infection with or without abnormal chest CT imaging findings. Mild COVID-19 was defined when there were mild clinical symptoms (such as slight fever and fatigue) and no features of pneumonia on imaging. Common COVID-19 was defined when there were symptoms of fever and respiratory symptoms (such as dry cough and running nose) and features of pneumonia on imaging.

### 2.2. Data Collection

The basic clinical information of patients were collected, including epidemiological history, clinical symptoms, number of SARS-CoV-2 nucleic acid-positive days, laboratory indicators including white blood cell count (WBC), neutrophil percentage (NEUT%), neutrophil absolute value (NEUT#), lymphocyte percentage (LYMPH%), lymphocyte absolute value (LYMPH#), platelet (PLT), alanine aminotransferase (ALT), aspartate aminotransferase (AST), AST/ALT, C-reactive protein (CRP), prothrombin time (PT), activation partial thrombin time (APTT), fibrinogen (FIB), D-dimers, and chest CT findings. All data were obtained from the Electronic Medical Record System of Shenzhen Hospital of Southern Medical University.

### 2.3. Detection of SARS-CoV-2-Specific Antibodies

SARS-CoV-2-specific IgM/IgG antibodies were detected on Time-Resolved Immuno-fluorescence Analyzer by Fluorescence immunochromatographic assay method (Lot: 20200214, Beijing Diagreat Biotechnologies Co., Ltd., Beijing, China). The cut-off value of IgM and IgG was 0.88 and 1.02, respectively. The results were shown as fluorescence intensity (Flu).

### 2.4. Statistical Analysis

All data were analyzed with SPSS 16.0 statistical software. Measurement data were displayed as the mean ± standard deviation (SD) and compared with an independent sample *t*-test. The count data was analyzed using a chi-square test. The correlation was analyzed by Spearman. A *P* value < 0.05 was considered statistically significant.

## 3. Results

### 3.1. Baseline Clinical Characteristics of Patients

A total of 52 patients with COVID-19 were included in this study. There were 38 adults, including 22 males and 16 females, with a median age of 36 years (range 19-66 years) (Table 1). In addition, there were 14 children, including 6 males and 8 females, with a median age of 6.33 years (range 0-15 years). In the adult group, 27 (71.05%) cases had a history of epidemiological exposure, and 9 (23.68%) cases had infections related to family clustering. However, 12 (85.71%) cases in the children group had a history of epidemiological exposure, and 7 (50%) cases had infections of family clustering. There was no statistically significant difference between the two groups in epidemiological exposure history and family cluster infections (*P* = 0.470 and *P* = 0.068, Table 1).

### 3.2. Symptoms

The most common symptoms of the patients were fever and cough (Table 1). In the adult group, 16 (42.11%) patients had fever, 13 (34.21%) patients had cough, 6 (15.79%) patients had throat discomfort, 4 (10.53%) patients each had fatigue and diarrhea, 3 (7.89%) cases each had headache and chest pain, and 1 (2.63%) case had runny nose and loss of taste (Table 1). Shortness of breath was not reported in the adult group. In the children group, there were 8 cases with fever (57.14%), 6 cases with cough (42.86%), 2 cases with expectoration (14.28), and 1 case each (7.14%) with fatigue, runny nose, shortness of breath, and diarrhea (Table 1). The children group had no symptoms such as headache, dizziness, and loss of taste. There was no statistically significant difference in symptoms between the adult and children groups. There were 5 cases (13.16%) of asymptomatic infections in the adult group and 4 cases (28.57%) of asymptomatic infections in the children group (Table 1).

### 3.3. Clinical Classification and Chest CT Performance

In the adult group, there were 29 patients with common infection (76.32%) and 9 patients with mild infection (23.68%) (Table 2). In the children group, there were 9 patients (64.29%) with common infection and 5 patients (35.71%) with mild infection. The difference between the two groups was not statistically significant (*P* = 0.390). In the adult group, 17 patients had ground-glass opacity (44.74%), 4 patients had local patchy opacity (10.53%), and 8 patients had multiple patchy ground glass opacities in bilateral lungs (21.05%), and none of the patients had consolidation (0%). In the children group, 3 patients had ground-glass opacity (51.43%), 1 patient had local patchy opacity (7.14%), 4 patients had multiple patchy ground glass opacity in bilateral lungs (25.57%), and 1 patient consolidation (7.14%). There was no statistically significant difference in CT results between the two groups (*P* = 0.812). In the adult group, there were 9 cases (23.68%) with unilateral lung involvement and 19 cases (50%) with bilateral lung involvement. However, in the children group, 3 cases (21.43%) had unilateral lung involvement and 6 cases (42.86%) had bilateral lung involvement. The difference between the two groups was also not statistically significant. Among the asymptomatic infections, 5 cases were adults (13.16%). Among them, 4 cases had ground-glass opacity and 1 case had multiple patchy opacities. Among the 4 cases of children (28.57%) with asymptomatic infections, there was 1 case with ground-glass opacity, 1 case with limited patchy opacity, 1 case with normal CT imaging, and 1 case with right lung middle lobe consolidation (Table 2). This case with right lung middle lobe consolidation was a female whose parents were diagnosed with COVID-19. She underwent chest CT examination due to the close contact history and later tested positive for SARS-CoV-2 nucleic acid.

### 3.4. Laboratory Findings

All 38 adults were positive for SARS-CoV-2 nucleic acid in nasal/pharyngeal swabs. However, 2/14 children cases were negative for SARS-CoV-2 nucleic acid in nasal/pharyngeal swabs, but tested positive for SARS-CoV-2-specific IgM and IgG. There were no statistically significant differences between the adult group and the children group in WBC, NEUT#, ALT, CRP, PT, APTT, FIB, D-dimer, and number of SARS-CoV-2-positive days (Table 3). However, the NEUT% of adults (57.98 ± 9.56) was significantly higher than that of children (38.36 ± 14.92) (*P* < 0.001). In addition, LYMPH%, LYMPH#, and PLT in adults were significantly lower than those of children, respectively (30.81 ± 9.06 vs. 50.84 ± 14.90, 1.71 ± 0.73 vs. 3.94 ± 2.12, and 214 ± 82.15 vs. 317 ± 115.17, respectively) (*P* < 0.001, *P* = 0.011, and *P* = 0.001, respectively). The normal range of ALT and AST is 0-45 U/L. In the adult group, patients with elevated ALT accounted for 15.79% (6/38), and patients with elevated AST accounted for 5.26% (2/38). However, in the children group, patients with elevated ALT accounted for 7.14% (1/14), and patients with elevated AST accounted for 28.57% (4/14). The AST and AST/ALT in the children group were higher than those in the adult group, which were 39.37 ± 18.04 vs. 26.52 ± 12.95 and 2.37 ± 1.16 vs. 1.16 ± 0.56, respectively. These differences between the two groups were statistically significant (*P* = 0.006 and *P* < 0.001, respectively) (Table 3).

### 3.5. Correlation Analysis

In addition, correlation analysis showed that there was no correlation of mild or common clinical classifications with LYMPH%, LYMPH#, NEUT%, PLT, AST, and AST/ALT (Table 4).

## 4. Discussion

The World Health Organization reports that an average of 20%-30% of toddlers and school-age children are affected by seasonal influenza outbreaks every year [13]. Unlike the common viral respiratory infections in children such as respiratory syncytial virus, adenovirus, rhinovirus, and influenza virus, the proportion of children infected with SARS-CoV-2 is not high [1, 2, 14]. A study involving 1099 patients with COVID-19 [14] found that there were only 9 children aged 0-14 years old, accounting for 0.9%, including 8 mild patients and 1 severe patient. A prospective multicenter observational cohort study in the United Kingdom recruited 651 children and young people (<19 years old) with SARS-CoV-2 [15]. The results showed that the all cause in-hospital case fatality rate was low at 1%, compared with 27% in the whole cohort of all ages (0-106 years) over the same time period. Another study from South Korea showed that 22% of 91 children were asymptomatic, 8.5% were diagnosed with symptoms, 66.2% had atypical symptoms, and 25.4% developed symptoms after diagnosis [16]. This means that symptom dependence screening fails to detect most cases of COVID-19 in children. Therefore, SARS-CoV-2 infection rate and fatality rate in children are significantly lower than in adults. Meanwhile, most children have mild or asymptomatic infections.

In this study, we retrospectively analyzed the clinical characteristics, laboratory indicators, chest CT findings, and number of days positive for SARS-CoV-2 nucleic acid in 14 children diagnosed with COVID-19 of which 5 (35.71%) had mild illness and 9 (64.29%) common presentation. Four of the 5 mild cases were asymptomatic of which 2 were negative for SARS-CoV-2 nucleic acid, but COVID-19 was confirmed by positive serology tests as well as radiological evidence of lung involvement. Moreover, in this study, 50% of children had a history of SARS-CoV-2 infections in family members, which was nearly twice that of adults (23.68%), consistent with previous studies [17–19]. These findings suggest that SARS-CoV-2 infection in children is the result of transmission between family members in the household. Therefore, if family members have traveled or lived in an epidemiological area, they need to self-isolate or adhere to preventative measures to reduce or prevent transmission to children. In addition, our findings highlight the importance of testing for SARS-CoV-2 in children by both nucleic acid and serological test methods as well as chest CT.

Among the 38 adult cases, 5/9 (13.16%) mild infections were asymptomatic which were lower than in children but this difference was not statistically significant. The reasons for the mild infection of children may include the following: (1) the quality or quantity of ACE2 receptors in children is different from that in adults [9]; (2) children rarely have comorbidities; (3) children are often infected with viruses when they are young, and the repeated exposure to viral infections may cross-protect them against SARS-CoV-2 [20, 21]; (4) children's immune system is not mature, and thus, there will be no severe inflammatory reaction after infection [21]; (5) children, especially those under 6 years old, have lymphocytes as high as 60%, which means that children have more lymphocytes to kill the virus. In addition, this study also found that even there were more severe imaging changes such as consolidation of the lung lobes in children; they could still be asymptomatic, suggesting that the inflammation caused by SARS-CoV-2 infection in children is lighter than that in adults. The reason may be related to the immature immune system of the children, which will not cause an excessive response of the immune system or severe inflammatory factor storm.

Wang et al. [22] observed the dynamic changes of lymphocyte counts in 33 COVID-19 patients (including 28 survivors and 5 dead patients) and found that most patients had significant lymphopenia. The lymphocytes of deceased patients gradually declined over time, indicating that the number of lymphocytes may be related to the severity and poor prognosis of COVID-19. It is also reported [23, 24] that CD4^+^ T cell depletion leads to a decrease in lymphocyte recruitment and cytokine production, leading to delayed immune-mediated SARS-CoV-2 clearance. In this study, we found that LYMPH% and LYMPH# in children were significantly higher than those in adults, while NEUT% in children were significantly lower than those in adults. These results are consistent with the study of DU et al. [25]. Chen et al. [26] compared the immune function of children and found that the levels of T cells, CD8^+^ T cells, and B cells in children were higher than those in adults. Higher levels of lymphocytes indicate higher capability to clear the virus.

Viral infection leads to a marked increase in PLT activity [27]. During the infection, the host inflammatory response leads to the release of PLT activation mediators, and PLTs can be activated by viral antigen-antibody complexes [28]. B lymphocytes also produce anti-PLT antibodies to certain viruses [29]. These processes that promote PLT activation will lead to increased PLT consumption and clearance, leading to PLT reduction. A decrease in PLT leads to an increased risk of death and the occurrence of acute respiratory failure [30]. In this study, we found that PLTs in children were significantly higher than those in adults. Therefore, in children, the PLT damage caused by the virus is less than that in the adult group. These results were consistent with the clinical classifications and lymphocyte levels of the two groups.

The systemic response syndrome caused by COVID-19 is closely related to the activation of natural immunity and cellular immunity triggered by SARS-CoV-2 infection [31]. Most SARS-CoV-2-infected pediatric cases are mild or asymptomatic, and only a small portion of pediatric cases will develop a multisystem inflammatory syndrome several weeks after SARS-CoV-2 infection or exposure, with severe cardiac complications, including shock, hypotension, and acute heart failure [32, 33]. Understanding postinfectious immune responses in pediatric SARS-CoV-2 infection is critical for treatment and prevention, especially with multisystem inflammatory syndrome in children. Here, we describe the impact of SARS-CoV-2 infection on children and adults, specifically the impact of viral infection on lymphocytes, platelet, and organ dysfunction. The virus can directly induce a variety of proinflammatory signals through toll-like receptors and promote the activation of T lymphocytes. Activated T lymphocytes attack infected cells, leading to apoptosis and necrosis. When T lymphocytes cannot completely eliminate the virus, they activate a variety of inflammatory signaling pathways, leading to macrophage activation and secondary inflammation. When more inflammatory cytokines are released, it eventually causes more cell damage and necrosis. This vicious circle causes damage not only to the lungs but also to multiple organs of the liver, heart, and kidneys [34]. Recent studies on COVID-19 have shown [13, 14] that mild-to-moderate aminotransferase elevations are very common in patients with COVID-19, although there have been no reports of acute liver failure. The proportion of liver injury in severe COVID-19 patients is significantly higher than that in mild COVID-19 patients [13, 35]. A study of 138 inpatients in Wuhan [22] showed that the ALT and AST levels of COVID-19 patients in ICU were higher than those not admitted to ICU. In addition, ALT levels greater than 40 IU/L are associated with patient mortality [36]. Furthermore, elevated AST and bilirubin levels may be associated with an increased risk of progression to respiratory failure and death. Although the available data is not clear whether an elevated liver enzyme level is an independent predictor of poor prognosis for COVID-19, elevated aminotransferases are common in patients of ICU and patients with mechanical ventilation, meaning that increased aminotransferases are more common in severely ill patients. Therefore, elevated aminotransferase is associated with the severity of the disease. Our study found no significant differences in ALT levels between the adult and children groups. However, the AST and AST/ALT ratios of children were higher than those of adults. About 28.57% children had higher AST than the normal limit of 45 U/L, while the proportion in adults was 5.26%. The tests on ALT and AST were all performed before the application of antiviral therapy. Thus, the increase in AST is not likely to be induced by the drug. This may, to some extent, indicate that children with SARS-CoV-2 infection are more prone to liver cell damage. However, due to the small sample size of this study and the lack of literature reports on liver function impairments in children and adults, we cannot yet make a definite conclusion.

This study has some limitations. First, the number of included children was relatively small. Second, anal swab results were not analyzed. Therefore, whether there is the possibility of fecal mouth transmission in the family is unclear. Third, more cases are needed to confirm whether there is liver injury after SARS-CoV-2 infection in children.

In summary, SARS-CoV-2 infections in children are mostly family clustering infections and are mostly mild and asymptomatic infections. Thus, timely isolation of family members with a history of epidemiological exposure is important for protecting children from SARS-CoV-2 infection. In addition, even asymptomatic children should undergo SARS-CoV-2 nucleic acid testing and chest CT to further determine whether they are infected. Lymphocyte reduction is common in adults, but not in children, which may relate to the physiological characteristics of children and may explain why children are less likely to be infected with SARS-CoV-2 than adults. Moreover, attention should be paid to SARS-CoV-2 infected children with elevated transaminases.

## Figures and Tables

**Table 1:** Patient characteristics, exposure status, and clinical symptoms.

	Total	Adults	Children	*P* value
Total	52	38 (73.08%)	14 (26.92%)	
Age (median) (years)		36	6.33	<0.001
Gender				
Male	28	22 (57.89%)	6 (42.85%)	0.344
Female	24	16 (42.11%)	8 (57.15%)	
Epidemiological exposure history	39	27 (71.05%)	12 (85.71%)	0.470
Family clustering infection	16	9 (23.68%)	7 (50%)	0.068
Symptoms				
Fever	24	16 (42.11%)	8 (57.14%)	0.335
Cough	18	13 (34.21%)	6 (42.86%)	0.524
Expectoration	2	0 (0%)	2 (14.28)	0.288
Dizziness	1	1 (2.63%)	0 (0%)	0.426
Headache	3	3 (7.89%)	0 (0%)	1.000
Fatigue	5	4 (10.53%)	1 (7.14%)	0.706
Throat discomfort	6	6 (15.79%)	0 (0%)	0.174
Runny nose	2	1 (2.63%)	1 (7.14%)	0.470
Loss of taste	1	1 (2.63%)	0 (0%)	0.426
Shortness of breath	1	0 (0%)	1 (7.14%)	0.269
Diarrhea	5	4 (10.53%)	1 (7.14%)	1.000
Chest pain	3	3 (7.89%)	0 (0%)	0.555
Asymptomatic infection	9	5 (13.16%)	4 (28.57%)	0.373

**Table 2:** Clinical classification and chest CT manifestations.

	Total	Adults	Children	*P* value
Total	52	38 (73.08%)	14 (26.92%)	
Clinical classification				
Mild	14	9 (23.68%)	5 (35.71%)	0.390
Common	38	29 (76.32%)	9 (64.29%)	
CT performance				0.812
Normal	14	9 (23.68%)	5 (35.71%)	0.386
Ground glass opacity		17 (44.74%)	3 (51.43)	0.226
Local patchy opacity		4 (10.53%)	1 (7.14%)	0.706
Patchy opacity of both lungs		8 (21.05%)	4 (25.57%)	0.842
Consolidation opacity		0 (0%)	1 (7.14%)	0.102
Unilateral lung involvement	12	9 (23.68%)	3 (21.43%)	1.000
Bilateral lungs involvement	27	19 (50%)	6 (42.86%)	0.647

**Table 3:** Comparison of laboratory results of COVID-19 patients in adults and children.

Items	Adults (*n* = 38) (Value ± SD)	Children (*n* = 14) (Value ± SD)	*P* value
WBC (×10^9^/L)	5.71 ± 2.16	6.92 ± 4.57	0.199
NEUT%	57.98 ± 9.56	38.36 ± 14.92	<0.001
NEUT# (×10^9^/L)	3.31 ± 1.46	2.77 ± 1.94	0.282
LYMPH%	30.81 ± 9.06	50.84 ± 14.90	<0.001
LYMPH# (×10^9^/L)	1.71 ± 0.73	3.94 ± 2.12	0.011
PLT (×10^9^/L)	214 ± 82.15	317 ± 115.17	0.001
ALT (U/L)	27.12 ± 15.61	18.51 ± 11.30	0.065
AST (U/L)	26.52 ± 12.95	39.37 ± 18.04	0.006
AST/ALT	1.16 ± 0.56	2.37 ± 1.16	<0.001
CRP (mg/L)	12.38 ± 21.10	4.42 ± 6.56	0.174
PT (s)	12.30 ± 1.10	12.42 ± 0.77	0.736
APTT (s)	36.83 ± 5.03	35.43 ± 6.46	0.414
FIB (g/L)	3.68 ± 0.96	4.77 ± 5.66	0.267
D-dimers (*μ*g/mL)	0.43 ± 0.28	0.47 ± 0.35	0.658
Number of SARS-CoV-2-positive days	4.92 ± 4.56	4.36 ± 5.27	0.708

WBC: white blood cell; NEUT%: neutrophil percentage; NEUT#: neutrophil absolute value; LYMPH%: lymphocyte percentage; LYMPH#: lymphocyte absolute value; PLT: platelet; ALT: alanine aminotransferase; AST: aspartate aminotransferase; CRP: C-reactive protein; PT: prothrombin time; APTT: activation partial thrombin time; FIB: fibrinogen.

**Table 4:** Correlation analysis.

	LYMPH%	LYMPH#	NEUT%	PLT	AST	AST/ALT
COVID-19 clinical classification	*r* = 0.020	*r* = −0.221	*r* = −0.066	*r* = −0.147	*r* = 0.125	*r* = 0.062
	*P* = 0.889	*P* = 0.116	*P* = 0.640	*P* = 0.298	*P* = 0.379	*P* = 0.661

LYMPH%: lymphocyte percentage; LYMPH#: lymphocyte absolute value; NEUT%: neutrophil percentage; PLT: platelet; ALT: alanine aminotransferase; AST: aspartate aminotransferase.

## Data Availability

The data used to support the findings of this study are available from the corresponding author upon request.
